# Deep Learning Approach for Screening Autism Spectrum Disorder in Children with Facial Images and Analysis of Ethnoracial Factors in Model Development and Application

**DOI:** 10.3390/brainsci11111446

**Published:** 2021-10-29

**Authors:** Angelina Lu, Marek Perkowski

**Affiliations:** Department of Electrical and Computer Engineering, Portland State University, Portland, OR 97207, USA; h8mp@pdx.edu

**Keywords:** autism, facial images, machine learning, deep learning, race and ethnicity, diagnosis, screening, neural network, bias, ASD

## Abstract

Autism spectrum disorder (ASD) is a developmental disability that can cause significant social, communication, and behavioral challenges. Early intervention for children with ASD can help to improve their intellectual ability and reduces autistic symptoms. Multiple clinical researches have suggested that facial phenotypic differences exist between ASD children and typically developing (TD) children. In this research, we propose a practical ASD screening solution using facial images through applying VGG16 transfer learning-based deep learning to a unique ASD dataset of clinically diagnosed children that we collected. Our model produced a 95% classification accuracy and 0.95 F1-score. The only other reported study using facial images to detect ASD was based on the Kaggle ASD Facial Image Dataset, which is an internet search-produced, low-quality, and low-fidelity dataset. Our results support the clinical findings of facial feature differences between children with ASD and TD children. The high F1-score achieved indicates that it is viable to use deep learning models to screen children with ASD. We concluded that the racial and ethnic-related factors in deep-learning based ASD screening with facial images are critical to solution viability and accuracy.

## 1. Introduction

Autism spectrum disorder (ASD) is a developmental disability that can cause significant social, communication, and behavioral challenges according to the Centers for Disease Control and Prevention (CDC). The estimated prevalence of ASD in the US is 1 in 59 children of ages 8 years and younger, and it is increasing [1]. However, significant and persistent racial and ethnic disparities exist in ASD prevalence as well as disparities in the accessibility to intervention and treatment services. In comparison to White children, children from racial and ethnic minority groups are less likely to be diagnosed with ASD and more likely to be misdiagnosed or suffer delayed diagnoses [2]. Although the combined estimated ASD prevalence was 16.8 per 1000 (1 in 59) children in 2018, it was significantly higher among non-Latino White children (17.2 per 1000) than among non-Latino African American children (16.0 per 1000), Latino children (14.0 per 1000), and Asian/Pacific Islander children (13.5 per 1000) [1].

These delayed or misdiagnoses for minority races result in a loss of opportunity in early intervention for children with ASD. Clinical results demonstrate that significant, longer-term gains are possible with early, comprehensive, and intensive intervention, and that these gains are evident in not only intellectual ability, language, and social behavior, but also in reductions in the severity of ASD symptoms [3]. In two cases, children who received the Early Start Denver Model (ESDM) therapy no longer met criteria for an ASD diagnosis [4]. A recent cost-comparison study of early intensive behavioral intervention in the Netherlands suggested that lifetime cost savings could be over EUR 1 million per individual if early intervention is initiated before 30 months of age [4]. These findings demonstrate how early identification and intensive ASD-specific intervention can improve long-term outcomes for children with ASD while emphasizing the need to extend this work further into underserved community settings to work toward improving outcomes for all children with ASD [4].

The major factors causing the disparity in ASD prevalence and delayed diagnoses in the U.S. are as follows [5]:

1. The subjectiveness in diagnosis: ASD is currently diagnosed by behavioral observation, and thus, only experienced clinicians can reliably diagnose ASD for children around 2 years old, with the mean age for diagnosis being 4–5 years [6].
2. Many families do not have access to experts/specialists, and the accessibility is even lower in underserved communities.
3. Lack of awareness and screening is also a problem, particularly in rural regions.
4. Additionally, children of racial and ethnic minority backgrounds who meet the criteria for ASD are less likely than White children to be diagnosed overall: they are more likely to be misdiagnosed.

Therefore, an objective, inexpensive, and easily comprehensible diagnosis or screening solution is imperative for supporting early intervention for the children with ASD of every family. To achieve this goal, in this research, we aimed to demonstrate that an early ASD screening method that uses solely children’s facial images with deep-learning is both viable and accurate.

**Figure 1:**
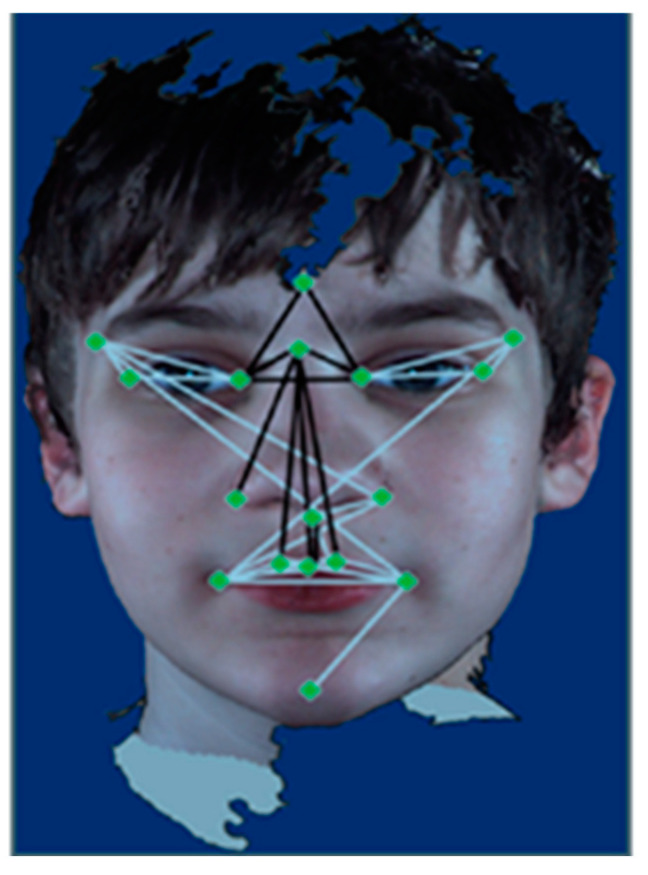
For boys with ASD compared with TD boys: the white lines are statistically significantly increased in length; the black lines are statistically reduced in length [7].

Clinical findings [7,8] have suggested that facial morphology is distinct between children with ASD and TD children. For example, boys with ASD may display certain facial phenotypic distinctions from TD boys (Figure 1) [7].

Whereas Aldridge et al. [7] used the Euclidean distance measurement for landmark points, Obafemi-Ajayi et al. [8] used the geodesic measurement, which also validated and extended Aldridge et al.’s [7] conclusions. Obafemi-Ajayi et al. [8] demonstrated that generalizing facial phenotypes is a viable biomarker for identifying ASD subgroups. They [8] concluded that the similarity of the results obtained in [7,8] was not dependent on measurement type (Euclidean vs. geodesic) or the cluster technique. This confirms that two-dimensional facial measurements provide replicable and important biomarkers in autism.

Additionally, girls with ASD displayed gender sex scores that were significantly lower (i.e., less feminine) compared to the control group [9].

**Figure 2:**
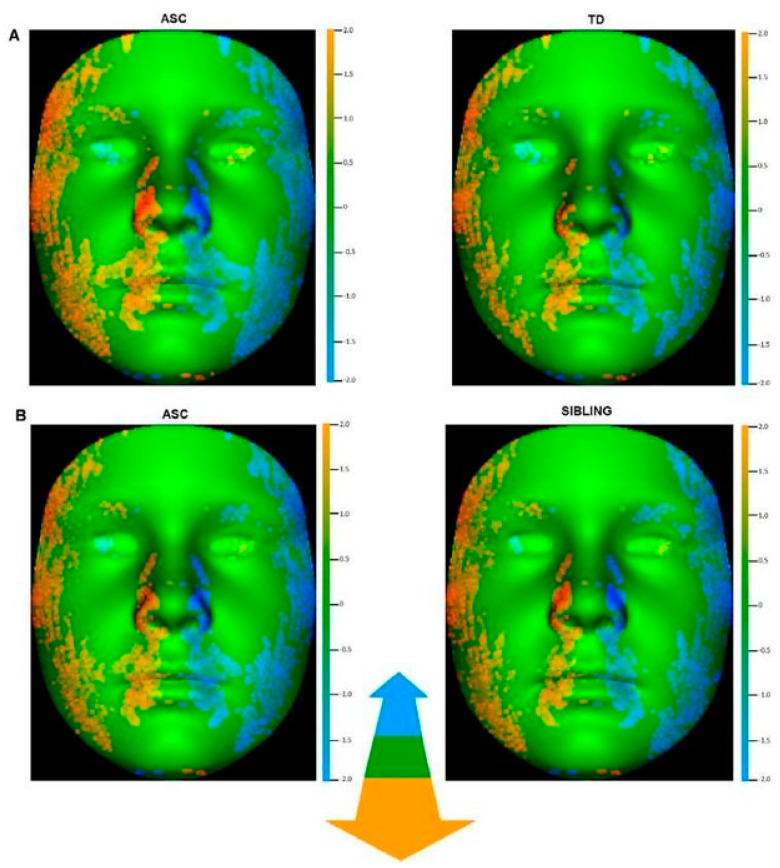
Comparison of depth facial asymmetry in mean original to mean mirrored form for (**A**) autistic children and TD children and (**B**) autistic children and sibling children. The color scale uses orange (or blue) to indicate where individual points on the mean original face is at least 2mm outside (or at least 2mm inside) corresponding points on the mean mirrored face. Figure 2A illustrate greater right-dominant depth asymmetry compared to TD children and Figure 2B illustrates greater right-dominant depth asymmetry in autistic children compared to siblings [10].

Boutrus et al. [10] reported an increased facial asymmetry in ASD, as shown in Figure 2.

Ozgen et al. [11] reported that morphological features are significantly increased in patients with autism.

Computer vision is a field of artificial intelligence (AI) that enables computers and systems to derive meaningful information from digital images, videos, and other visual data—taking actions or making recommendations based on such information. If AI enables computers to think, computer vision enables them to see, observe, and understand [12]. According to the systematic reviews of published studies about computer vision in ASD by de Belen et al. [13] and Rahman et al. [14], no published study has used computer vision technology with deep learning to diagnose ASD using a facial image dataset that met review eligibility criteria. It was emphasized that the current state of computer vision methods applied to ASD research is not well established, amid increasing evidence that suggests that computer vision techniques have a strong impact on autism research [13]. Almost all previous research focused on functional magnetic resonance imaging (fMRI), facial expression or emotion, eye movement tracking, or behavior-related analysis.

We think that this lack of reported advances in facial-image-based machine learning solutions for screening ASD is due to the unavailability of high-fidelity ASD facial image datasets. The only publicly available dataset is the Kaggle ASD Children Facial Image Dataset [15]. However, the author of the Kaggle dataset stated that the ASD images were all collected via an internet search from online autism Facebook groups and other sources. Thus, the quality of the images in the dataset is cause for concern [16]. ASD diagnosis confirmation accuracy is imperative when applying deep learning for ASD facial image classification. We found that the Kaggle dataset mixes images from different races with a ratio of about 89% White children to 11% children of color. We discuss why the race factor is critical and such mix of races in the Kaggle dataset is problematic. We use the Kaggle dataset as an illustration only to draw proper attention to its implications.

We recognize that a gap exists in applying computer vision using facial images to screen children for ASD.

Our method, using clinically diagnosed ASD children’s images from the Elim Autism Rehabilitation Center, specialized in early ASD intervention and rehabilitation for children diagnosed with ASD, obtained high accuracy in the screening of children for ASD with deep learning and bridges the gap in this field.

## 2. Materials and Methods

### 2.1. Datasets

#### 2.1.1. East Asia ASD Children Facial Image Dataset (East Asian Dataset)

The East Asian dataset contains 1122 images evenly split between children with ASD and TD children from the same race. We collected about 600 facial images from the Elim Autism Rehabilitation Center, which specializes in children with ASD and is headquartered in Shandong, China. About 8000 children with ASD have completed their intervention and therapy programs in this rehabilitation center since its establishment in 2000. We obtained the support of Elim Autism Rehabilitation Center management, through which consent and privacy agreements were obtained from the families of the children to use their children’s images specifically to support this research. We also augmented this dataset with 561 images of TD children from several kindergartens and elementary schools in China. All of the images are from children aged between 2 and 12 years and of the same race. This is the dataset we used for solution proposal and accuracy conclusions.

#### 2.1.2. Kaggle Autism Facial Dataset (Kaggle Dataset): The Only Publicly Available ASD Facial Image Dataset

This dataset consists of 2936 facial images evenly split between children with ASD and TD children [17]. The original dataset contained 3014 images [15], which posed obvious problems, as indicated in [16]. As the contributor also stated that he could not obtain any ASD images from institutions or verifiable sources, all the images in the Kaggle dataset were the results of internet search [16]. We used the dataset in [17], which contained 2936 images after removal of obviously wrong images. This dataset contains about 89% White children and 11% children of color. We only used this dataset to illustrate the impact of the race factors in facial-image-based, deep-learning development.

### 2.2. Method

In recent years, deep convolutional neural networks (CNNs) have been used extensively in computer vision, showing powerful discriminative capabilities while maintaining high performance levels [18]. As deep learning networks have established themselves as a promising model for facial recognition and CNNs have been used as the deep learning tool in almost all facial recognition systems [19], our research focused on a deep-learning-based solution. In a recent comparative study of popular deep-learning architectures for facial recognition, Gwyn [20] reported that VGG16/VGG19 showed the highest accuracy levels of image recognition; as such, we further focused our study using VGG16-based deep learning.

Transfer learning is a machine learning method where a model developed for one task is reused as the starting point for a model on a second task, and is a popular approach in deep learning. Visual Geometry Group (VGG) is a CNN model proposed by Simonyan and Zisserman [21] that achieved 92.7% accuracy, placing it in the top-five in test accuracy on ImageNet, a dataset of over 14 million images belonging to 1000 classes [22].

VGGFace is a facial image dataset that contains 2.6 million images of 2622 people contributed by the Visual Geometry Group [22].

**Figure 3:**
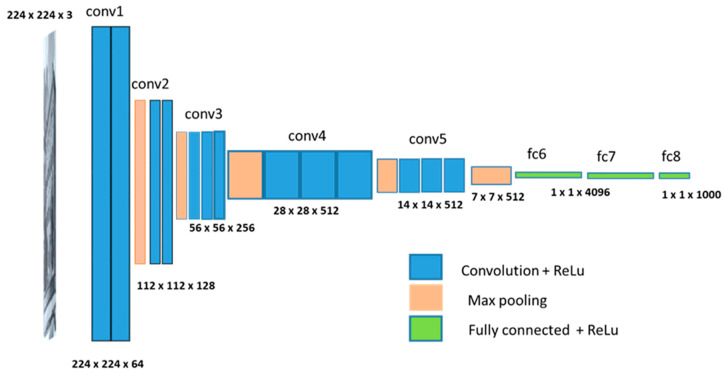
VGG 16 architecture.

Tensorflow is an end-to-end open-source platform for machine learning. It has a comprehensive, flexible ecosystem of tools, libraries, and community resources [23]. Keras is the high-level API of Tensorflow [24]. Keras-VGGFace is an Oxford VGGFace implementation using Keras Functional Framework v2+ [24]. A VGG16 model pre-trained with VGGFace is provided in Keras-VGGFace. Thus, VGG16 was adopted for this research as the pre-trained model for transfer learning. Figure 3 shows the VGG16 architecture.

Our research was conducted in two major focus areas:

1. The feasibility and quality of applying deep learning in the detection of ASD in children using 2D facial images
2. Understanding the significance of race factors in ASD detection or diagnosis using deep learning and facial images

Let us revisit some of the metrics for model accuracy measurement. True positive (TP) is a prediction where the model correctly predicts the positive class. True negative (TN) is the prediction where the model correctly predicts the negative class in a binary classification. False positive (FP) is the prediction where the model incorrectly predicts the negative class. False negative (FN) is the prediction where the model incorrectly predicts the positive class. Classification accuracy (CA) is the rate of correct classifications.

- CA = (TP + TN)/(TP + FN + FP + TN)
- PRECISION = (TP)/(TP + FP)
- RECALL = (TP)/(TP + FN)
- F1-SCORE = 2 * (PRECISION * RECALL)/PRECISION + RECALL)

#### 2.2.1. Feasibility and Classification Accuracy Study of Applying Deep Learning to Detect ASD in Children Using 2D Facial Images

In this feasibility and accuracy study, we used the East Asian dataset for model training and verification because the ASD facial images in this dataset are from clinically diagnosed children from a single race.

We first used the Orange visual ML platform [25] for model development and architecture selection in terms of performance as measured by F1-scores and classification accuracy (CA). The Orange platform is a convenient ML result visualization tool suitable for fast feasibility studies.

**Figure 4:**
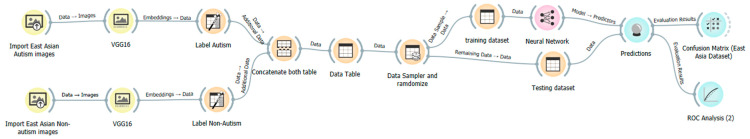
VGG16 transfer-learning-based deep-learning model pipeline architecture for the East Asian dataset training and classifications using the Orange ML platform (screen shot).

Figure 4 describes the model training and testing pipeline architecture. We used VGG16 as the pre-trained model for image embedding. The neural network model is composed of two hidden layers before the classifier. We used Adam (stochastic gradient-based) as the optimizer [26] and rectified linear unit (ReLU) [27] as the activation function in the hidden layers. We applied the standard 10-fold cross validation method. The dataset was split into 80% and 20% for training and testing, respectively.

#### 2.2.2. Classification Improvement with Tensorflow/VGGFace

Based on the results of the feasibility experiment with the Orange platform, we determined that the VGG16 transfer-learning-based neural network [28] is viable for classification of 2D ASD images, showing quality performance. Our next experiment involved improving the classification accuracy. We decided to use Tensorflow/VGGFace with the East Asian dataset to fine-tune the model to achieve best performance.

We used a Keras-VGGFace implementation with the VGG16 pre-trained model [24] and froze 70% of the base model layers. Different Keras learning rates and other parameters were adjusted, such as trainable layers, during model training. With various experiments, we decided to append two (FC8 and FC9) hidden dense layers with 100 neurons each for ASD feature training and a dropout rate of 0.25 for the FC8 layer to reduce potential overfitting. Table A1 shows the architecture and layer details.

The training dataset contained 882 images, and the validation dataset contained 230 images, evenly split between ASD and non-ASD classes.

#### 2.2.3. Understanding the Significant Impact of Race Factors on Deep-Learning-Based ASD Detection with Facial Images

Recent studies demonstrated that most of the commercial facial analysis software and algorithms are biased against certain categories of race and ethnicity [29]. As we were developing facial image-based ASD detection algorithms, understanding the racial impact was critical to providing accurate and reliable deep learning and facial image-based solutions. More importantly, when applying facial image-based machine learning approaches to screening or diagnosis in medical fields, classification errors due to race factors in the model should be eliminated.

Race factors tend to be overlooked by researchers and readers. For example, we noticed that a recently published study [30] used the Kaggle ASD facial dataset entirely to derive its deep-learning solution and accuracy. We would like to illustrate the importance of race factors and discuss this topic from the anthropometrics perspective to draw proper attention from interested readers and authors to this matter.

The analysis was focused on the misclassifications of Black and East Asian children.

As mentioned earlier, although the Kaggle dataset is of low quality, and it is questionable whether it can be used to support any claims on the validity or accuracy of deep-learning solutions, we could use the Kaggle dataset to illustrate how race-related factors can impact the deep-learning solution for ASD detection. The Kaggle dataset contains facial images from different races. By visually examining the images in the dataset, we determined that it contains roughly 89% White children, 4.29% Black children, 1.1% East-Asian-looking children, and about 5.7% of other children of color, similar to Musser’s [17] reported 10:1 ratio for White children vs. children of color.

We used the same Orange platform model pipeline in Figure 4 for the race factor analysis experiments.

The first experiment (***Exp-1***) used the Kaggle dataset to train and test the model and observe the misclassifications for Black children.

The second experiment (***Exp-2***) used the East Asian test dataset, which was also used in Section 2.2.1 to test the model trained in ***Exp-1***. The purpose of the experiment was to observe the misclassifications for East Asian children.

The third experiment (***Exp-3***) used the same model architecture and configuration in Figure 4, but the model was trained by combining the Kaggle and East Asian datasets. In this experiment, we significantly increased the East Asian training data from about 1.1% to 28.44% to understand if by increasing the East Asian percentage in the Kaggle dataset, the model could yield better classification accuracy compared with ***Exp-2***.

The race distribution for the combined dataset is shown in Table 1.

The fourth experiment (***Exp-4***) added additional race labels to the combined dataset.

For all the previous experiments, there were only two class labels. This was suitable for the East Asian dataset since it is a single-race dataset. However, the new, combined dataset contained different races, with two large groups being White and East Asian. We decided to implement additional classification labels to understand how different races with significant anthropometric differences mixed in the same dataset could affect the classification accuracy.

**Figure 5:**
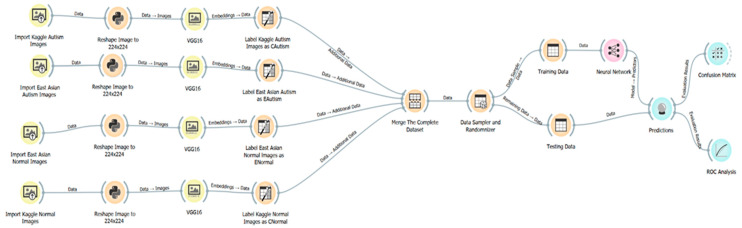
Pipeline architecture (Orange platform screen shot) with the four classification target classes described in Table 2.

Figure 5 depicts the modified pipeline architecture from Figure 4. We labeled the combined Kaggle and East Asian dataset target classes from 2 classes (*Autism* vs. *Normal*) to 4 classes. Because nearly 89% of the Kaggle dataset was White children, for simplicity, we added the letter “C” to the beginning of previous class labels of the images in the Kaggle dataset. We added the letter “E” as the initial to the previous class labels of the images in the East Asian dataset. The expanded target classes were *CAutism, CNormal, EAutism* and *ENormal,* as shown in Table 2.

Figure 5 depicts the dataflow pipeline architecture with four target classes.

## 3. Results

### 3.1. Evaluation of Deep-Learning Solution Viability and Accuracy with the East Asian Dataset

#### 3.1.1. Results for Section 2.2.1

Table 3 shows the results of the deep learning model performance using the Orange platform and the East Asian dataset described in Section 2.2.1, Figure 4.

The VGG-16 embedding followed by the neural network model with two hidden layers achieved a classification accuracy of 93.3% and F1 score of 0.928, and thus, it proved to be feasible to use this VGG-based deep-learning solution to detect ASD using facial images. The confusion matrix for the model is shown in Table 4.

#### 3.1.2. Improved Classification Results from the Fine-Tuned Tensorflow/VGGFace-Based Deep-Learning Model with the East Asian Dataset

This deep-learning model architecture is described in Section 2.2.2 and Table A1.

**Figure 6:**
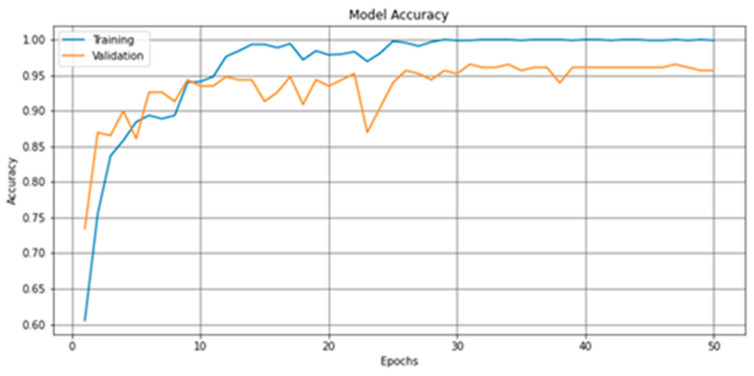
The accuracy graph produced by the deep-learning method described in Section 2.2.2 and Table A1.

**Figure 7:**
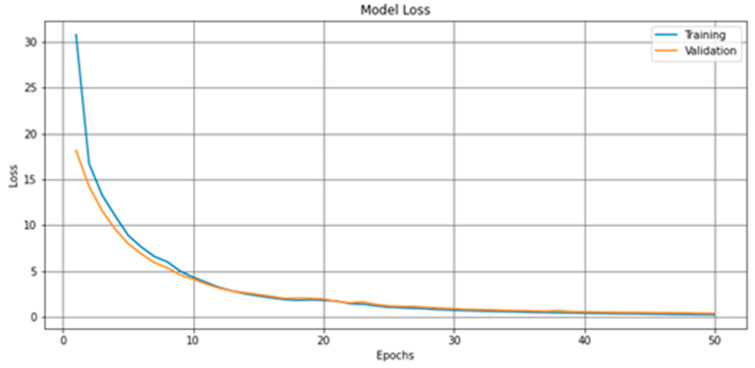
The loss graph produced from the deep-learning model described in Section 2.2.2 and Table A1.

The model achieved the best Val_accuracy in the 31st epoch at 0.957, as indicated in Figure 6. Figure 7 is the model loss graph.

The model achieved a 0.95 F1-score and 95% CA on the East Asian testing dataset, an improvement of about 2% over the model implemented with the Orange visual platform. Table 5 and Table 6 provide the confusion matrix, F1-score, and CA.

The 0.95 F1-score and 95% CA achieved in our experiment with the East Asian dataset suggest that our deep learning-based solution for screening for ASD with facial images is not only viable but also highly accurate.

### 3.2. Evaluation of the Results from Racial Factor Related Experiments Described in Section 2.2.3

#### 3.2.1. Evaluation of the Results of ***Exp-1***, ***Exp-2***, and ***Exp-3*** in Section 2.2.3

We used the Kaggle dataset to train and test the same model architecture and configuration in Figure 4 in Section 2.2.1. The purpose was to gain insights into how the racial factors impact the classification.

Table 7 is the confusion matrix from ***Exp-1*** where the model was trained and tested with the Kaggle dataset.

**Figure 8:**
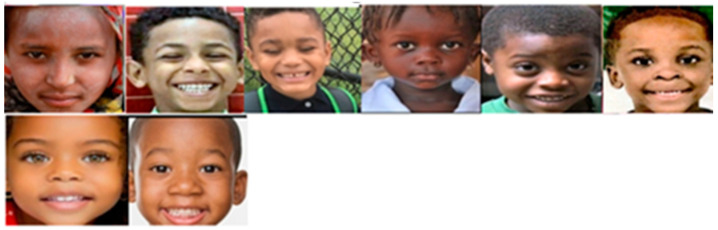
Six (top row in the figure) out of the eight TD Black children’s images in the Kaggle test dataset were misclassified as *Autism*.

By manually examining the 26 FP images and all of the 141 test images labeled *Normal* in Table 7, we found that there were only eight Black children’s images among the 141 *Normal* images. However, six of the eight *Normal* Black children’s images were misclassified as *Autism,* which is an FP Rate as high as 75% (6/8 = 75%) for Black children. Figure 8 shows the eight *Normal* Black children. Six (images in the top row) out of the eight images were misclassified.

Table 8 shows that Black children in the whole Kaggle dataset are poorly represented (~4.25% in total).

The confusion matrix in Table 9 is from ***Exp-2,*** where the same model in ***Exp-1*** was used. However, the East Asian testing dataset was used for testing. We can see that 98 out of 113 *Normal* East Asian test images were misclassified as *Autism,* yielding an FP rate of 86.7%.

There were only 32 East-Asian-looking images in the Kaggle dataset. Once again, this race was poorly represented in the Kaggle dataset.

The results from both ***Exp-1*** and ***Exp-2*** indicate high FP rates for the minorities, with 75% and 86.7% FP rates for Black children and East Asian children, respectively.

The confusion matrix from Table 10 is the result of ***Exp-3***. In ***Exp-3,*** we used the same model architecture as in ***Exp-1*** and ***Exp-2***, but we enhanced the training dataset by combining both Kaggle and East Asian datasets. We effectively increased East Asian training images from about 1.1% to 28.44% of the total training dataset. We still used the same East Asian testing dataset as in ***Exp-2*** to test the model, resulting in 27 FP cases compared to 98 in Table 9.

By increasing the East Asian images in the Kaggle dataset, we observed significant improvement in the East Asian FP rate, from 86.44% (98/113 = 86.7%) to 23.9% (27/113 = 23.9%). However, compared with the FP rate of 6.67% (8/120 = 6.67%) in Table 4, we still observed a significant difference. Table 11 and Table 12 provide the comparisons.

#### 3.2.2. Evaluation of the Results from ***Exp-4*** with Race Group Labeling

Referring to Table 2 and Figure 5, we changed the target classes from two to four, i.e., instead of *Autism* and *Normal*, we had *CAutism*, *CNormal*, *EAutism*, and *Enormal* as class labels.

The confusion matrix in Table 13 is from ***Exp-4***.

We next focused on the FP cases for *ENormal* labeled images that were the East Asian non-autism images. There was a total of 103 East Asian non-autism test images (labeled as *ENormal*). Among the 103 *ENormal*-labeled images, 80 were classified correctly as *ENormal*; 11 images were misclassified as *CAutism*, implying that these 11 images were more compatible with the Kaggle autism class criteria for the model trained. There were also three *ENormal*-labeled images that were misclassified as *CNormal*. Summing all the misclassified cases for *ENormal* test images, we observed a similar FP rate of 22.3% (23/103 = 22.3%) compared with the FP rate of 23.89% from ***Exp-3*** in Table 11.

Table 14 includes all of the 11 cases where *ENormal*-labeled images were misclassified as *CAutism*.

The first 10 images of the misclassification cases would otherwise be classified as *ENormal* correctly if we provided the race information at the time of prediction because the probability of being *ENormal* was the second highest. Knowing that the facial image was an East Asian subject eliminated the possibility of being *CAutism*. The only outlier was the image N497, with a 5% probability of being *ENormal* following a 57% probability of being *CAutism* and 38% probability of being *CNormal*. Because N497 was labeled as *Enormal*, it could be neither *CAutism* nor *CNormal*. Therefore, if we applied the known race information indicating that the image was an East Asian subject, the *ENormal* (5%) probability for this image should still have prevailed because *EAutism* was not likely (a less than 0.1% probability), and *CNormal* or *CAutism* should have been ruled out.

In conclusion, all of the 11 misclassifications could be corrected because the highest probability would be *ENormal* (East Asian normal) when *CAutism* or *CNormal* were excluded from the prediction pool based on knowing the test image’s race information.

In Table 15, there are three *ENormal*-labeled images misclassified as *CNormal*. Table 15 shows the probability distribution of the three cases.

With similar analyses of these three cases, we inferred that these three cases should also be classified as *ENormal* correctly once we applied the known East Asian race information in the prediction.

Let us compare the confusion matrices in Table 4 and Table 13.

For the model trained with the East-Asian-only dataset (Table 4), there were eight normal cases misclassified as autism, for a ratio of 8/120 (=6.67%).

For the model trained with the combined datasets (Table 15), for the East Asian normal cases (labeled as *ENormal*), there were a total of 23 cases misclassified as *CAutism* (11), *CNormal* (3), and *EAutism* (9), or as not *ENormal*. The ratio was 23/103 (=22.3%). However, if we eliminated the impact of *CNormal* (3) and *CAutism* (11) as we discussed, a total of 14 impossible cases resulted when the race information was known, yielding a ratio of 9/103 (=8.74%).

These results, considering known race information in the prediction, were significantly better and closer to the model trained with a single race in Section 3.1.1 (FP rates of 6.67% vs. 8.74% for the East Asian testing images). FN cases for the East Asian test images could be analyzed similarly. Note that we only used the Kaggle dataset to qualitatively illustrate the race factors, as we labeled the Kaggle dataset as a “single” race for simplicity while 11% of its images were actually from other races.

We performed additional experiments (***Exp-5*** and ***Exp-6***) by removing all other races except White children from the Kaggle dataset to form a single-race dataset. We cleaned up the dataset further by removing obvious poor-quality images. The Kaggle dataset size was reduced from 2936 to 1910 images. We repeated ***Exp-3*** in ***Exp-5*** and ***Exp-4*** in ***Exp-6*** using the new combined dataset. Similar results to ***Exp-3*** and ***Exp-4*** were obtained for the *ENormal* class FP rate, as indicated in Table A2, Table A3 and Table A4 in Appendix A. We noticed a ~6% difference due to the Kaggle dataset cleanup, but compared to the 6.67% FP rate in ***Exp-1***, 23.9% in ***Exp-3***, and 17.6% in ***Exp-5***, the improvement had no material impact on the conclusion (see Table A2, Table A3, Table A4 and Table A5 for details).

## 4. Discussion

### 4.1. Regarding Race Factors in Facial Image Based Diagnostic Solutions including ASD Detection

#### 4.1.1. Understanding the Anthropometrics within the Context of Diagnosis Based on Facial Phenotype Distinctions

Facial features are generally different among different races. For instance, “African-Americans have statistically shorter, wider, and shallower noses than Caucasians” [31].

Anthropometrics show the racial morphometric differences in the craniofacial complex [32]. Based on carefully defined facial landmark points, 25 measurements on head and face were captured to examine three racial groups (i.e., North American White, African American (Black), and Chinese). Farkas identified several differences in these three groups. For example, the Chinese group had the widest faces; the main characteristics of the orbits of the Chinese group were the largest inter-canthal width. Furthermore, the soft nose was less protruding and wider in the Chinese group, and they had the (relatively) highest upper lip in relation to mouth width, etc. [33].

Virdi et al. [34] described comparative anthropometry in relation to African Americans and North American Whites (NAWs). Virdi et al. [34] detected significant differences between Kenyans and North American Whites (NAWs). Some of the significant differences were, for example, in forehead height (~5 mm greater for men, ~4.5 mm for women), nasal height (reduced by ~4 mm in men, ~3 mm in women), nasal width (8–9 mm greater), upper lip height (>3 mm), and eye width (greater by ~3 mm). All vertical measurements obtained were significantly different compared with NAWs. The study [34] concluded that facial anthropometric measurements of NAWs show clear differences compared with the Kenyan population. Race variability should always be considered during diagnosis and treatment planning of orthognathic or craniofacial reconstructive treatment. Treating subjects from different race groups using normative anthropometric data from another group for comparison may be misleading and inaccurate [8,35,36,37].

Virdi et al. [34] did verify that anthropometric measurements of Caucasian populations are invalid when applied to the Kenyan population. They recommended that accurate and applicable data be used in diagnosis and treatment planning for each race group.

**Figure 9:**
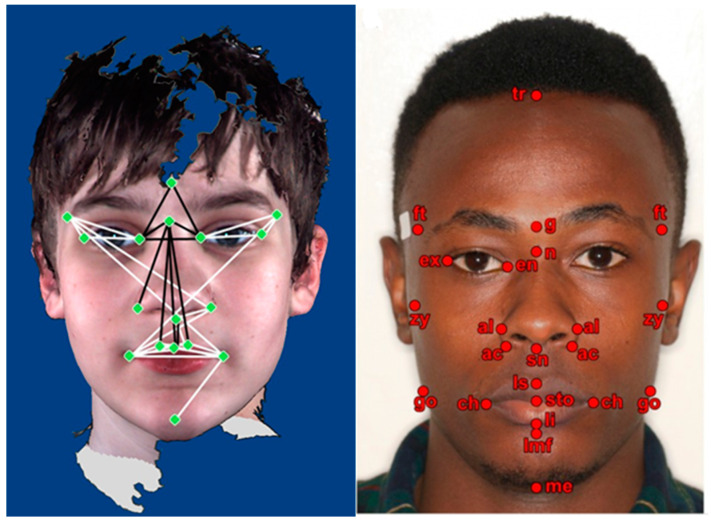
Image on the left [7] indicates significant facial landmark changes in boys with ASD. Image on the right [34] indicates the facial feature landmarks used in a comparative anthropometry difference study in relation to Kenyan-Africans, African Americans, and North American Whites.

In Figure 9, the image on the left identifies the significant facial landmark feature changes in boys with ASD [7]. All the white lines are statistically significantly increased, while all the black lines are statistically significantly reduced in boys with ASD relative to TD boys. The image on the right in Figure 9 identifies the landmark features used in the study by Virdi et al. [34] to identify the anthropometric differences in relation to Kenyan-Africans, African Americans, and North American Whites.

Statistically, normal Kenyan African women’s eye width (ex-en) in Table 16 is 33.7 mm, whereas that of NAWs is 30.7 mm, with a *p*-value < 0.001 (A small *p*-value, for example, less than 0.05 (typically ≤ 0.05), indicates a statistically significant difference. In this case, the clinically significant difference was set at ±3 mm). For African Americans (AAs), the inter-canthal distance en-en compared to NAWs was significantly longer, at 34.4 mm vs. 31.8 mm, with a *p*-value < 0.001 (Table 16) [34].

Fang et al. [38] concluded that the greatest interethnic variability in facial proportions exists in the height of the forehead. More pronounced differences among ethnic groups are also present in measurements of the eyes, nose, and mouth. There is no significant difference between sexes in the neoclassical facial proportions.

Some of these significant differences also fall into ASD-related facial landmark feature changes.

As facial-image-based computer vision relies on facial anthropometric data to find the abnormalities or alterations to detect ASD, we had to confirm that our dataset was constructed correctly, without mixing races with significantly different facial anthropometric measurements. Ozgen et al. [11] also concluded that as ethnicity can influence the prevalence of ASD morphological abnormalities, homogenous datasets should be utilized.

#### 4.1.2. Findings from the Experiments Regarding the Race Impact on Deep-Learning- Based ASD Screening with Facial Images

We revisit the results from the experiments in Section 3.2, and the analysis in Section 3.2.2. We drew the following conclusions:

1. The neural network deep-learning model trained with the East Asian dataset achieved an F1-score of 0.928 and CA of 92.8% with the Orange platform.
2. We achieved a high F1-score of 0.95 and a CA of 95% with the Tensorflow/VGGFace-based deep learning model on the East Asian dataset (see Table A1 for architecture). The results suggest that it is viable to use deep learning solutions for high-accuracy ASD screening.
3. Due to the race factor impact in the Kaggle dataset, the model trained with the Kaggle dataset generated 75% and 86.7% FP rates for Black and East Asian test images, respectively.
4. When combining the Kaggle and East Asian datasets for training, which effectively increased the training images for East Asian children, we observed an improved FP rate for the East Asian test dataset, from 86.7% to 23.9%. However, compared with the 6.67% FP rate from the model trained and tested with the East Asian dataset, the single-race dataset indicated in Table 4 and Table 11, the 23.9% FP rate was still much worse, although each experiment had almost an equal number of training images for East Asian children. We think that this result is due to anthropometric differences amongst different races, for example, Whites vs. East Asians. It is possible that one race’s normal facial anthropometric measurements can fall into another race’s abnormal facial anthropometric measurements or vice versa, resulting in mistaken classifications, as in the cases shown in Table 13 and Table 14, where normal East Asian images labeled as *ENormal* were misclassified as *CAutism*. The comparison in Figure 9 and the anthropometry in Table 16, e.g., ex-ex/en-en lengths [34], indicates the possibility of one race’s facial anthropometric changes due to ASD falling into another race’s normal ranges, or vice versa. The analysis of Table 13 and Table 14 from the ***Exp-4*** results confirms that this occurred when we added the labels to the combined dataset with race group information.

### 4.2. Brief Discussion of Video-Based Deep-Learning Approach and 2D Facial Image-Based Approach

The standard approaches to diagnosing autism spectrum disorder (ASD) evaluate between 20 and 100 behaviors and take several hours to complete [39]. To make this approach easier and faster, several researchers reported using videos with machine learning to accelerate and automate the process [39,40,41,42]. These proposed video-based approaches use tablets or other devices that can capture the child’s behaviors, for example, eye gaze, or responses to stimuli, while the child is watching the specially designed movie clips or engaging in activities. The machine learning model then provides the classification results. We can categorize these ASD detection mechanisms as behavior phenotype-based approaches [42]. The proposed ASD detection method using deep learning with 2D facial images can be categorized as a facial-phenotype-based approach. The video-based detection solution is reported to achieve >90% accuracy [39] and significantly reduce the screening time. However, for many families in the world, it is more expensive than solutions that simply use a 2D picture for at-home ASD risk assessment. It still requires a certain amount of time for the child to focus on the video, which may be difficult for some children with ASD. As race factors are critical to the facial-image-based solution, further studies need to be conducted on the video-based approach to understand if cultural differences can be factors that cause bias toward certain ethnic groups [43]. For example, the content of the movie clips or the toys used for the activities may be culture-specific. We also need to understand if culture/ethnic group-specific models need to be developed similarly to the M-CHAT per each country’s cutoff scores [44]. To further increase the reliability of both video and image-based solutions, more research can be conducted to combine the solutions to detect both facial phenotype and behavior phenotype distinction.

### 4.3. Recommendations

High accuracy and high reliability are critical in medical-related diagnosis or screening. Proper race-related consideration is imperative in proposing and developing accurate facial-image-based deep-learning solutions.

To achieve the highest possible accuracy and eliminate interference due to differences in facial anthropometrics from different races, we recommend that race-specific models be developed to eliminate an impact or bias from “other race” factors on the reliability and accuracy of the deep-learning models based on 2D facial images for medical diagnosis or screening.

Pertaining specifically to ASD screening for children with our facial-image-based deep learning solution, we recommend that homogenous race facial image datasets be utilized for algorithm development, solution viability, and accuracy claims.

## 5. Conclusions

The high classification accuracy of 95% and F1-score of 0.95 obtained by our deep learning model trained with the East Asian dataset indicates that it is viable to use children’s facial images as a low-cost solution to screen for ASD to achieve early intervention objectives.

This study bridges the gap of applying computer vision in ASD screening ASD in children using their facial images.

The results of this study support the clinical findings of facial feature differences between children with ASD and TD children.

We think that this computer vision solution will help to address major causes of racial disparity in ASD diagnosis or screening, such as the subjectiveness in screening or diagnosis [45], the difficulty in access to professional medical services, and the financial obstacles families face in many regions and especially impoverished countries. Future studies can focus on transforming the solution into a user-friendly mobile application to allow families to simply use a cellphone to take a picture and receive an immediate screening result with high accuracy. Lightweight deep learning models, as described in [46,47], could further accelerate the productization of the solution in this study.

Our findings support authors’ conclusions that racial differences must be considered in related medical treatment or diagnosis [34,35,36,48].

We also concluded that for facial-image-based deep-learning solutions, race-specific datasets should be built for model development to eliminate errors in classification due to anthropometric differences among races. Furthermore, the race information of the subject to be diagnosed or classified should be known as a prerequisite to use the applicable model in ASD diagnosis or screening.

Further research should also be conducted to combine both image- and video-based approaches into one solution to enable the detection of both behavior phenotype and facial phenotype distinctions in ASD to further eliminate misclassifications.

## Figures and Tables

**Table 1:** Race distribution in the combined dataset.

Race/Ethnicity	Percentage	Count
Black	3.10%	126
East Asian	28.44%	1154 ^1^
Other children of color	4.07%	165
White	64.39%	2613
Total	100.00%	4058

^1^ The East Asian count includes 1122 from the East Asian dataset and 32 from the Kaggle dataset.

**Table 2:** Using four classification target classes with additional race information preceding the label.

Dataset	Subset	Label ^1^
Kaggle	Autism	*CAutism*
	Non-Autism	*CNormal*
East Asian	Autism	*EAutism*
	Non-Autism	*ENormal*

^1^ Image labels preceded with *C* or *E* indicate the image belongs to the Kaggle or the East Asian dataset, respectively.

**Table 3:** Results for deep learning feasibility experiment with Orange platform and East Asian dataset.

Model	UAC	CA	F1	Precision	Recall
Neural Network	0.983	0.933	0.928	0.932	0.923

**Table 4:** Confusion matrix for neural network model trained and tested with the East Asian dataset.

		Predicted		
		Autism	Normal	Σ
**Actual**	Autism	96	8	104
	Normal	8	112	120
	Σ	104	120	224

**Table 5:** Confusion matrix produced by the deep-learning model described in Section 2.2.2 and Table A1.

		Predicted		
		Autism	Normal	Σ
**Actual**	Autism	112	3	115
	Normal	8	107	115
	Σ	120	110	230

**Table 6:** Classification report for the method in Section 2.2.2 and Table A1 (reproduced from screenshot).

	Precision	Recall	F1-Score	Support
Autism	0.93	0.97	0.95	115
Normal	0.97	0.93	0.95	115
Accuracy			0.95	230
Macro average	0.95	0.95	0.95	230
Weighted average	0.95	0.95	0.95	230

**Table 7:** Confusion matrix from ***Exp-1*** (model trained and tested with the Kaggle dataset).

		Predicted		
		Autism	Normal	Σ
**Actual**	Autism	127	26	153
	Normal	26	115	141
	Σ	153	141	294

**Table 8:** Black children image count in the Kaggle dataset.

Kaggle Dataset	Total Count	Black Children Image Count	Black Children Images Percentage of Total
Images labeled as ASD	1468	58	3.95%
Images Labeled as non-ASD	1468	68	4.63%

**Table 9:** Confusion matrix from ***Exp-2*** (model trained with the Kaggle dataset but tested against the East Asian test dataset).

		Predicted		
		Autism	Normal	Σ
**Actual**	Autism	106	7	113
	Normal	98	15	113
	Σ	204	22	226

**Table 10:** Confusion matrix from ***Exp-3*** (model was trained with the combined Kaggle and East Asian datasets but tested against the East Asian test dataset).

		Predicted		
		Autism	Normal	Σ
**Actual**	Autism	84	29	113
	Normal	27	86	113
	Σ	111	115	226

**Table 11:** FP rates for each of the experiments with the same deep-learning architecture in Figure 4.

Experiment Section	Training Dataset	Test Dataset	% of East Asians in Training Dataset	*Normal* Images in the Test Dataset	FP Cases	FP Rate
Section 2.2.1	East Asian	East Asian	100%	120	8	6.67%
Section 2.2.3. ***Exp-2***	Kaggle	East Asian	1.1%	113	98	86.73%
Section 2.2.3. ***Exp-3***	Combined ^1^	East Asian	28.44%	113	27	23.89%

^1^ The combined training dataset is composed of both Kaggle and East Asian training datasets.

**Table 12:** Model performance for each of the experiments.

Experiment Section	Training Dataset	Test Dataset	CA	F1	Precision	Recall
Section 2.2.1	East Asian	East Asian	0.933	0.928	0.932	0.923
Section 2.2.3. ***Exp-2***	Kaggle	East Asian	0.513	0.667	0.507	0.973
Section 2.2.3. ***Exp-3***	Combined ^1^	East Asian	0.752	0.750	0.757	0.743

^1^ The combined training dataset is composed of both Kaggle and East Asian training datasets.

**Table 13:** Confusion matrix from ***Exp-4*** (model trained and tested with the combined dataset with additional race group labels).

Predicted						
Actual		*CNormal*	*ENormal*	*EAutism*	*CAutism*	Σ
	*CNormal*	231	3	0	55	289
	*ENormal*	3	80	9	11	103
	*EAutism*	1	6	105	8	120
	*CAutism*	63	11	6	219	299
	Σ	298	100	120	293	811

**Table 14:** Probability distribution for the 4 targeted classes for the 11 cases where *ENormal* was misclassified as *CAutism*.

Test ID	Image Name	Label	Misclassified as	Prediction Probabilities for Each Target			
				*CNormal*	*ENormal*	*EAutism*	*CAutism*
82	N691	*ENormal*	*CAutism*	0.000	0.180	0.010	0.810
276	N600	*ENormal*	*CAutism*	0.000	0.050	0.000	0.950
310	M-4	*ENormal*	*CAutism*	0.000	0.060	0.020	0.920
541	N583	*ENormal*	*CAutism*	0.000	0.360	0.000	0.640
462	N728	*ENormal*	*CAutism*	0.010	0.400	0.140	0.450
476	N730	*ENormal*	*CAutism*	0.000	0.130	0.000	0.860
413	N168	*ENormal*	*CAutism*	0.000	0.470	0.010	0.510
648	N716	*ENormal*	*CAutism*	0.050	0.140	0.010	0.800
38	N335	*ENormal*	*CAutism*	0.000	0.330	0.060	0.600
541	N583	*ENormal*	*CAutism*	0.000	0.360	0.000	0.640
618	N497	*ENormal*	*CAutism*	0.380	0.050	0.000	0.570

**Table 15:** Probability distribution for the four targeted classes for the three cases where *ENormal* was misclassified as *CNormal*.

Test ID	Image Name	Label	Misclassified as	Prediction Probabilities for Each Target			
				*CNormal*	*ENormal*	*EAutism*	*CAutism*
82	N691	*ENormal*	*CNormal*	0.830	0.150	0.010	0.010
276	N600	*ENormal*	*CNormal*	0.700	0.160	0.010	0.130
310	M-4	*ENormal*	*CNormal*	0.700	0.160	0.010	0.130

**Table 16:** Facial anthropometrics comparison of Kenyan Females with African Americans and North American Whites.

Kenyan Women’s Faces					
	KM Mean (n = 36)	NAW (SD) (n = 200)	*p* Value	AA (SD) (n = 50)	*p* Value
Vertical measurements					
Forehead height II tr-n	67.5 (2.9)	63.0 (6.0)	<0.001 *	67.1 (5.9)	0.693
Nasal height n-sn	47.6 (3.1)	50.6 (3.1)	<0.001 *	48.8 (3.7)	0.114
Lower face height sn-me	69.5 (4.8)	64.3 (4.0)	<0.001 *	71.5 (5.2)	0.061
Upper lip height sn-sto	24.0 (2.5)	20.1 (2.0)	<0.001 *	24.5 (3.0)	0.435
Lower lip height sto-sl	20.7 (1.1)	17.8 (4.7)	<0.001 *	20.2 (2.4)	0.163
Horizontal measurements					
Intercanthal distance en-en	32.1 (1.4)	31.8 (2.3)	0.225	34.4 (0.5)	<0.001 *
Eye width ex-en	33.7 (1.5)	30.7 (1.2)	<0.001 *	32.2 (2.0)	0.087
Biocular width ex-ex	94.4 (4.9)	87.8 (3.2)	<0.001 *	92.9 (5.3)	0.185
Nasal width al-al	40.7 (3.7)	31.4 (2.0)	<0.001 *	40.1 (3.2)	0.411
Mouth width ch-ch	52.0 (4.0)	50.2 (3.5)	0.012	53.6 (4.0)	0.073

* Clinically significant difference set at ±3 mm [34].

## Data Availability

Data are available on request due to privacy restrictions. The data presented in this study are available on request from the corresponding author. The images are not publicly available because the image owners (ELIM Autism Rehabilitation Center management and parents of the children) only permitted their use in this study. However, upon request, we can supply the image imbedding data of each image used in this study.

## Appendix A

**Table A1:** CNN based Deep Learning model architecture described in Section 2.2.1 and result reported in Section 3.1.2.

Layers (Type)	Output Shape
input	224, 224, 1
ConV_1 × 2	224, 224, 64
Pool1	112, 112, 64
ConV_2 × 2	112, 112, 128
Pool2	56, 56, 128
ConV_3 × 3	56, 56, 256
Pool3	28, 28, 256
ConV_4 × 3	28, 28, 512
Pool4	14, 14, 512
ConV_5 × 3	14, 14, 512
Pool5	7, 7, 512
flatten	25088
Fc6 (Dense)/fc6 reLU	4096
Fc7(Dense)	4096
dense (Dense)	100
Dropout	100
dense_1 (Dense)	100
classifier (Dense)	2

**Table A2:** Confusion matrix from ***Exp-5. Exp-5*** repeated ***Exp-3*** with the Kaggle dataset replaced with the cleaned Kaggle dataset described in Section 3.2.2.

Confusion Matrix (Trained with the Combined (Cleaned) Kaggle and East Asia Datasets and Tested with East Asian Test Dataset				
		Predicted		
		Autism	Normal	Σ
**Actual**	Autism	101	19	120
	Normal	22	103	125
	Σ	121	122	245

**Table A3:** Confusion matrix from ***Exp-6***. ***Exp-6*** repeated ***Exp-4*** with the Kaggle dataset replaced with the cleaned Kaggle dataset as described in Section 3.2.2.

Confusion Matrix: Model trained with Kaggle and East Asia Datasets Combined with 4 Target Classes as in Table 13. The Cleaned Kaggle Dataset Only Contains White Children.						
		Predicted				
		CNormal	Enormal	EAutism	CAutism	Σ
**Actual**	CNormal	168	5	1	48	222
	ENormal	2	102	9	9	122
	EAutism	0	7	105	6	118
	CAutism	44	10	5	115	174
	Σ	214	124	120	293	636

Note: After eliminating the *CAutism* and *CNormal* misclassification cases for *ENormal* in Table A4, the FP rate improved from 16.4% to 7.38% (very close to the 6.67% FP Rate in ***Exp-1***), indicating the significance of eliminating the race factor.

**Table A4:** Probability distribution for the 4 targeted classes for the 11 cases in Table A3 where *ENormal* was misclassified as *CAutism* (9) and *CNormal* (2).

Image Name	Label	Mis-Classified as	Prediction Probabilities for Each Target			
			*CNormal*	*ENormal*	*EAutism*	*CAutism*
**N756**	***ENormal***	*CAutism*	0.48	0.01	0.01	0.51
N510	*ENormal*	*CAutism*	0.00	0.03	0.01	0.96
N187	*ENormal*	*CAutism*	0.00	0.45	0.00	0.54
N476	*ENormal*	*CAutism*	0.00	0.00	0.00	1.00
N279	*ENormal*	*CAutism*	0.10	0.38	0.00	0.52
N686	*ENormal*	*CAutism*	0.07	0.00	0.00	0.93
N495	*ENormal*	*CAutism*	0.27	0.00	0.00	0.73
N316	*ENormal*	*CAutism*	0.00	0.02	0.00	0.98
N689	*ENormal*	*CNormal*	0.47	0.23	0.00	0.30
N317	*ENormal*	*CNormal*	0.00	0.36	0.00	0.64
N170	*ENormal*	*CAutism*	0.00	0.01	0.00	0.99

**Table A5:** Results comparison regarding the effect of Kaggle dataset cleanup.

**Experiment**	**Model Trained with**	**Version of Kaggle Dataset Used in the Combined Dataset**	**Test Dataset**	**FP Rate**	**Difference**
***Exp-1***	East Asian	N/A	East Asian	6.7%	
***Exp-3***	Combined Dataset of Kaggle and East Asian datasets	Original version with mixed races and invalid images; Dataset size is 2936	East Asian	23.9%	6.3%
***Exp-5*** (repeat ***Exp-3***)	Combined Dataset of Kaggle and East Asian datasets	Cleanup version with only White with removal of other identifiable invalid images; Dataset size is 1910	East Asian	17.6%	
**Experiment**	**Model Trained with**	**Version of Kaggle Dataset Used in the Combined Dataset**	**Test Dataset**	**FP Rate (East Asian)**	**Difference**
*****Exp-4*****	Combined Dataset of Kaggle and East Asian datasets	Original version with mixed races and invalid images; Dataset size is 2936	Combined	22.3%	5.9%
***Exp-6*** (repeat ***Exp-4***)	Combined Dataset of Kaggle and East Asian datasets	Cleanup version with only White with removal of other identifiable invalid images; Dataset size is 1910	Combined	16.4%	
